# Anterior cervical discectomy and fusion with zero-profile versus stand-alone cages for two-level cervical spondylosis: A retrospective cohort study

**DOI:** 10.3389/fsurg.2022.1002744

**Published:** 2022-11-02

**Authors:** Guanzhang Mu, Hao Chen, Haoyong Fu, Shijun Wang, Hailin Lu, Xiaodong Yi, Chunde Li, Lei Yue, Haolin Sun

**Affiliations:** ^1^ Department of Orthopedic, Peking University First Hospital, Beijing, China; ^2^ Department of Rehabilitation Medicine, Peking University First Hospital, Beijing, China

**Keywords:** anterior cervical discectomy and fusion (ACDF) surgery, cervical spine, disc herniation, outcome, stand-alone cage, zero-profile cage, two-level cervical spondylosis

## Abstract

**Objective:**

To assess the mid-long-term clinical and radiological outcomes of zero-profile (ZP) compared with stand-alone (ST) cages for two-level anterior cervical discectomy and fusion (ACDF).

**Methods:**

We included 77 patients (39 women and 38 men) who underwent two-level ACDF between May 5, 2016, and May 5, 2020, and who were followed up for at least 1 year. The subjects were divided into the ST (*n* = 38) and ZP (*n* = 39) group. For the evaluation of functional status, Japanese Orthopedic Association (JOA), Neck Disability Index (NDI), and Visual Analogue Scale (VAS) scores were used. Additionally, radiological outcomes and procedure complications were observed at final follow-up.

**Results:**

Both groups had excellent clinical outcomes at the final follow-up. There were no significant intergroup (ZP vs. ST) differences in the fusion rate (91.02% vs. 90.79%, *P *> 0.05) and postoperative dysphagia (15.4% vs. 2.6%, *P* = 0.108). However, the disc height at the final follow-up in the ZP group was higher than that in the ST group (6.86 ± 0.84 vs. 6.17 ± 1.03, *P* = 0.002). The ZP group accomplished a lower loss of cervical lordosis (18.46 ± 4.78 vs. 16.55 ± 4.36, *P* = 0.071), but without reaching statistical significance.

**Conclusion:**

ACDF with either ZP or ST cages turns out to be a dependable strategy for two-level ACDF in terms of clinical results. However, compared with the ST, the ZP cage may achieve a significantly lower loss of disc height.

## Introduction

Anterior cervical discectomy and fusion (ACDF) decompresses compressed spinal cord and nerve root, recovers physiological lordosis, and provides stability and anatomical height of the intervertebral disc. The technique was initially developed for treating cervical spondylosis, and the efficacy and safety of using ACDF for treating patients with radiculopathy and myelopathy are excellent (1–3). Anterior cervical plate technique has been reported to increase the operation time and the risk of postoperative dysphagia (4). In contrast, non-plate interbody implants, the stand-alone (ST) cage and zero-profile (ZP) cage have been widely used for ACDF surgeries. The ST cage in ACDF has positive effects in terms of recovery of physiological disc height, rapid improvement of the cervical lordosis, and facilitation of joint fusion (1, 5). However, ST cages have been reported to cause complications like cage migration, subsidence, and revision surgery (6, 7). By contrast, immediate postoperative stabilization the core advantage of a ZP cage and an ACP system (8). Unlike the latter, the ZP cage has an additional anchoring function, which ensures less protrusion in front of the vertebral body, thereby placing less compression on the esophagus and ultimately decreasing the risk of postoperative dysphagia (9, 10). Due to the ability to overcome these disadvantages of the ST cage and ACP construct, the use of zero-profile cages in ACDF is gradually increasing.

For one-level ACDF, the ZP cage contributes to the improvements in neurological function and cervical lordosis, similar to those of the ST cage but with lower risk of implant failure (1, 10, 11). However, the number of current clinical articles comparing multi-level ACDF using ZP with that using ST is rare. Here, we aimed to explore comprehensive radiological and clinical outcomes in patients who had undergone two-level consecutive ACDF using ZP cages compared with those in whom ST cages were used.

## Materials and methods

### Patients and study design

This retrospective cohort study was conducted at Peking University First Hospital and received an approval from the local ethics committee (No. 2021133). Between May 5, 2016, and May 5, 2020, 166 patients were screened, of whom 77 (66.2% women and 33.8% men) were eligible. Intervertebral disc degeneration screening prior to surgery involved x-ray radiography, computed tomography (CT), and magnetic resonance imaging (MRI). We retrospectively collected patients aged at least 18 years with two-level consecutive ACDF between C3 and C7. ALL patients reported an intractable radiculopathy or myelopathy that lasted at least 6 weeks and was refractory to nonsurgical therapies, including physical therapy and anti-inflammatory drugs. Participants were excluded if they experienced nerve compression as a result of an acute trauma, tumor, infection, or other reason. Participants were also excluded if they had a history of cervical surgery or revision surgery. Depending on the device used for ACDF, the subjects were divided into the stand-alone cage (ST) group (*n* = 38) or the zero-profile (ZP) group (*n* = 39).

### Surgical procedure

To expose the surgical segments, the right transversal incision was made in the supine posture of the patient. In most cases, the right transversal incision is sufficient. A distractor was utilized to access the lesioned intervertebral space after fluoroscopy had revealed that the area required decompression. During the process of decompression, nucleus pulposus and osteophytes were removed until we got satisfied decompression. When all the foregoing procedures were finished, ZP or ST cages were implanted. Intraoperative x-ray was used to verify that disc height and cervical alignment were restored, and to check all implants were in good position. For about 8 weeks after surgery, the patients were required to wear a cervical collar.

### Clinical outcomes and assessment

We collected the data regarding the surgical levels, operative time, blood loss, and patient's weight and height from the anesthesia records. All the participants had been instructed to fill out evaluations at the time of surgery, at 3-month intervals, and at the final follow-up. For the evaluation of functional status, Japanese Orthopedic Association (JOA), Neck Disability Index (NDI) (12), and the neck Visual Analogue Scale (VAS) pain scores were used. The prevalence of dysphagia was determined by utilizing the Bazaz system (Table 1) (13).

**Table 1:** Bazaz grading system for dysphagia.

Symptom severity	Liquid food	Solid food
None	None	None
Mild	None	None
Moderate	None or rare	Occasionally (only with specific food)
Severe	None or rare	Frequent (majority of solids)

### Radiological outcomes

Cervical curvature, disc height, and subsidence were investigated based on the lateral cervical x-ray radiographs. The imaging examinations were completed after the operation (within 4 days), at postoperative 3 months, and every 6 months thereafter until the final follow-up. The Cobb angle of C2–C7 (between the lower endplate of C2 and the lower endplate of C7) was used to examine the cervical lordosis (14). The disc height (DHI) was defined as the distance between the highest section of the cephalad vertebra's lower endplate and the closest region of the caudal vertebra's upper endplate (14). Subsidence was defined as an intervertebral height reduction of more than 2 mm relative to that immediately after surgery (14). A solid fusion was present if the following features were observed: the lateral x-ray observation of cervical hyperextension curve showed that there was no abnormal activity between the fusion segment and the spinous process; x-ray images of cervical vertebra showed that the two ends of the fusion cage were tightly combined with the upper and lower contact surfaces of the vertebral body without transparent band; and bone connection and trabecular formation appeared on x-ray and CT. The presence of any two of the above conditions was marked as interbody fusion (15). To compensate for discrepancies in radiological measures, three qualified investigators separately examined radiological outcomes at least three times.

### Statistical analyses

SPSS statistical software version 20.0 (IBM corp., Armonk, NY) was used for all analyses and calculations. In cases of continuous variables, the mean and standard deviation (SD) were used. Chi-square tests or Fisher's exact tests were used to compare categorical variables. To assess intergroup differences in numerical variables, the Student *t* test or Mann-Whitney *U* test was employed depending on compliance with a normal distribution or not. We utilized paired *t* tests to investigate the differences within the same group between different time points. Statistical significance level was set at *P* < 0.05.

## Results

### Participants’ baseline data and functional status: VAS, NDI, and JOA

A total of 77 patients (66.2% women and 33.8% men) were eventually included in this study. There were 39 patients (78 segments) in the ZP group and 38 patients (76 segments) in the ST group, of the 154 analyzed segments for which at least 1 year follow-up (mean 17.2 months, range 12–26 months) was completed. Sex, age, BMI, surgical levels, operative time, and intraoperative blood loss were not significantly different between the two groups (Table 2).

**Table 2:** Demographic outcomes of patients and clinic outcomes.

Parameters	Total	ZP group (*n* = 39)	ST group (*n* = 38)	*P* value
Patients (*n*)	77	39	38	
Sex (male/female)	39/38	13/26	13/25	0.94
Age (years)	54.2 ± 7.1	54.2 ± 7.0	54.1 ± 7.2	0.64
BMI (kg/m^2^)	25.6 ± 5.8	25.1 ± 3.0	26.1 ± 7.8	0.72
Surgical levels				
C3–C5	8	4	4	
C4–C6	33	15	18	
C5–C7	36	20	16	
Operative time (min)	95.52 ± 38.2	96.7 ± 38.9	94.4 ± 38.1	0.87
Blood loss (ml)	86.8 ± 9.6	88.7 ± 8.4	85.2 ± 10.4	0.11
Follow-up period (months)	17.23 ± 4.9	16.89 ± 4.5	17.56 ± 4.3	0.43

BMI, body mass index; ST, stand-alone cages; ZP, zero-profile cages.

The descriptive statistics illustrating the clinical outcomes of the included patients are listed in Table 3. The two groups had a fairly equivalent neurologic enhancement and pain alleviation according to VAS, NDI, and JOA at all time points. However, compared with preoperative state, these indicators improved markedly at any time point following surgery (*P* < 0.05), both in the ZP group and in the ST group.

**Table 3:** Patients’ functional status.

Variable	ZP group (*n* = 39)	ST group (*n* = 38)
JOA scores		
Preoperative	7.7 ± 2.3	7.8 ± 2.1
Postoperative at 3 months	14.1 ± 0.7*	14.3 ± 0.6*
At final follow-up	14.9 ± 0.8*^,^**	14.8 ± 0.8*^,^**
NDI scores		
Preoperative	18.2 ± 2.9	17.9 ± 3.0
Postoperative at 3 months	11.0 ± 1.2*	11.0 ± 1.8*
At final follow-up	10.4 ± 1.6*^,^**	10.6 ± 1.6*^,^**
VAS neck scores (0–10)		
Preoperative	6.3 ± 1.4	6.5 ± 1.6
Postoperative at 3 months	2.2 ± 0.7*	2.3 ± 0.7*
At final follow-up	1.7 ± 0.5*^,^**	1.8 ± 0.6*^,^**

JOA, Japanese Orthopedic Association; NDI, Neck Disability Index; VAS, Visual Analogue Scale of neck; ST, stand-alone cages; ZP, zero-profile cages.

*P < 0.05, compared with preoperative value (paired *t* test within-group).

**P < 0.05, compared with postoperative 3 months (paired *t* test within-group).

### Radiological outcomes: C2–C7 Cobb angle, DHI and fusion rate

**Figure 1:**
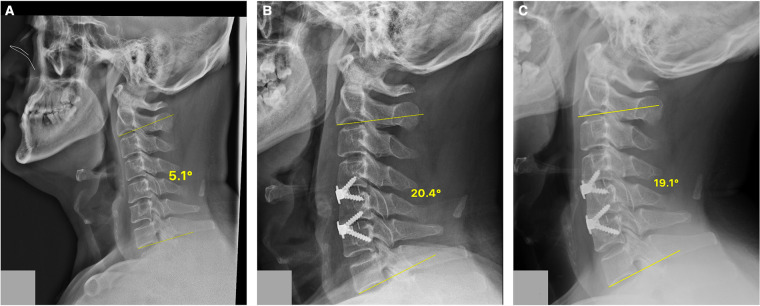
A case of the ZP group. Cervical lateral radiographs of a 47-year-old woman from the ZP cage group showing the C2–C7 Cobb angle at different time points. (**A**) 5.1° preoperatively; (**B**) 20.4° postoperatively; (**C**) 19.1° at the final follow-up. ZP, zero-profile cage.

**Figure 2:**
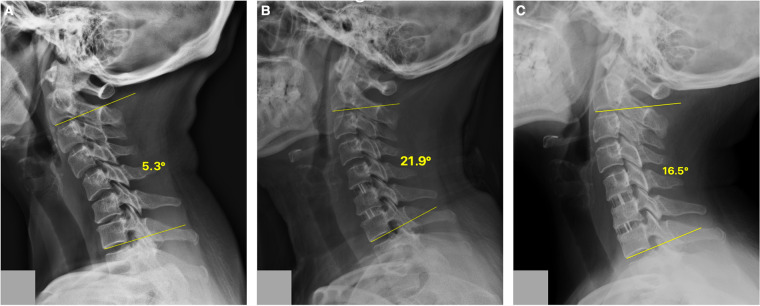
A case of the ST group. Cervical lateral radiographs of a 56-year-old woman from the ST group showing the C2–C7 Cobb angle at different time points. (**A**) 5.3° preoperatively; (**B**) 21.9° postoperatively; (**C**) 16.5° at the final follow-up. ST, stand-alone cage.

**Figure 3:**
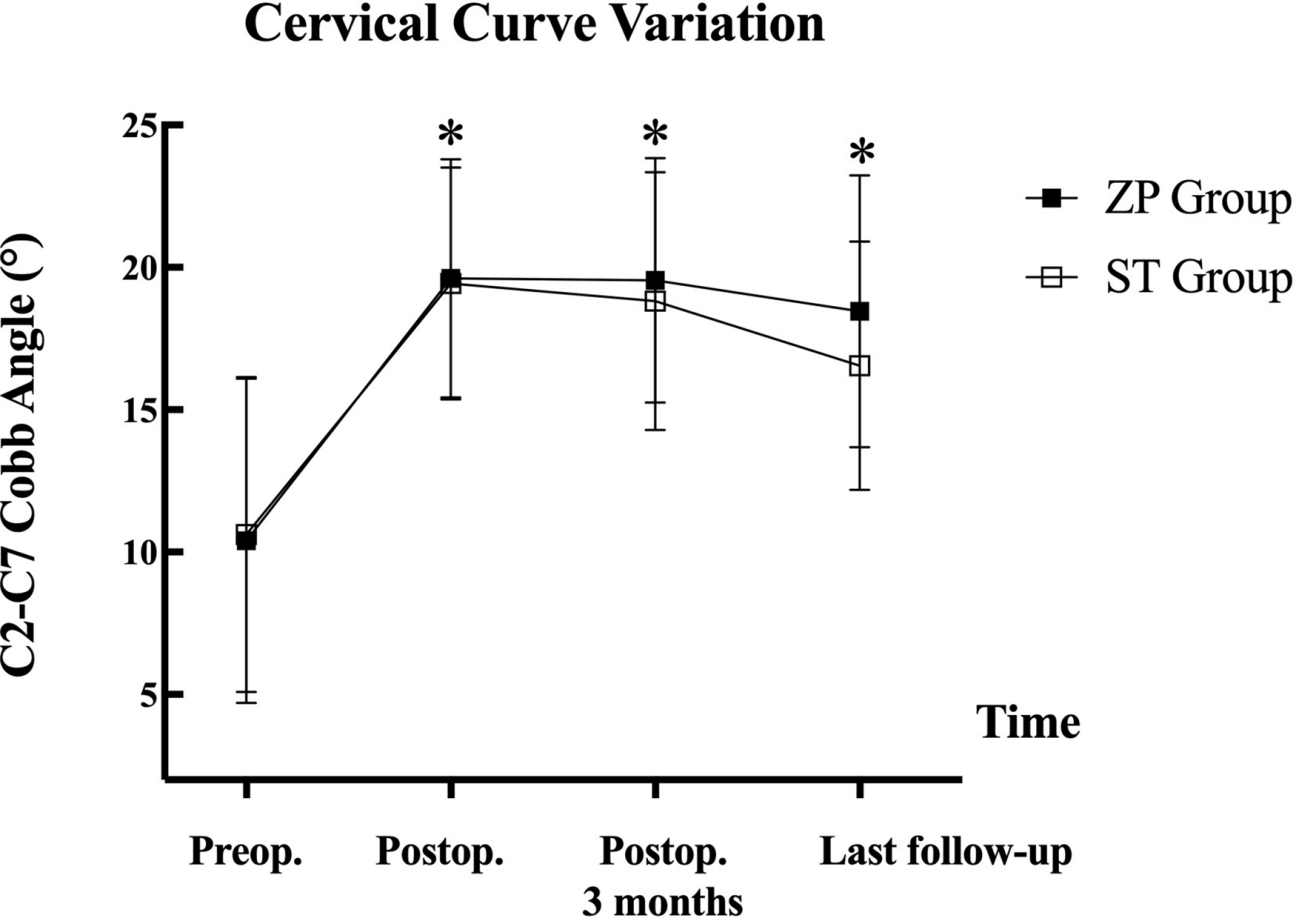
Variation of C2–C7 Cobb angle during the follow-up for ZP group and SC group. ZP, zero-P cage; ST, stand-alone cage; Preop, preoperative; Postop, postoperative. *P < 0.05, compared with preoperative value.

The descriptive statistics illustrating the radiological outcomes of the patients are listed in Table 4. The C2–C7 Cobb angle was used to measure cervical curvature. Although the Cobb angle of the ST and ZP groups declined with time, the C2–C7 Cobb angle in all patients was substantially improved at any point after treatment (*P* < 0.05, Table 4). At 3 months following surgery, the C2–C7 Cobb angle exhibited various degrees of decrease when compared to that immediately after the treatment. Figures 1, 2 depict the changes in C2–C7 Cobb angle over time. However, there were neither significant differences between various follow-up time points within the same group nor significant differences between the two groups at the same follow-up time points (Figure 3).

**Table 4:** Patients’ radiological outcomes.

Variable	ZP group (*n* = 39)	ST group (*n* = 38)
C2–C7 Cobb angle (°)		
Preoperative	10.40 ± 5.69	10.62 ± 5.53
Postoperative	19.61 ± 4.19*	19.43 ± 4.07*
Postoperative at 3 months	19.55 ± 4.30*	18.81 ± 4.53*
At final follow-up	18.46 ± 4.78*	16.55 ± 4.36*
DHI (mm)		
Preoperative	5.58 ± 1.38	5.76 ± 1.30
Postoperative	7.12 ± 1.48*	7.03 ± 1.51*
Postoperative at 3 months	6.98 ± 0.67*^,^**	6.78 ± 0.65*^,^**
At final follow-up	6.86 ± 0.84*^,^****	6.17 ± 1.03*^,^***
Fusion rate [*n*/*N* (%)]	71/78 (90.02)	68/76 (90.79)

DHI, disc height; ST, stand-alone cages; ZP, zero-profile cages.

* *P* < 0.05, compared with preoperative value (paired *t* test within-group).

** *P* < 0.05, compared with postoperative (paired *t* test within-group).

*** *P* < 0.05, compared with postoperative 3 months.

**** *P* < 0.05, significant difference between the two groups.

**Figure 4:**
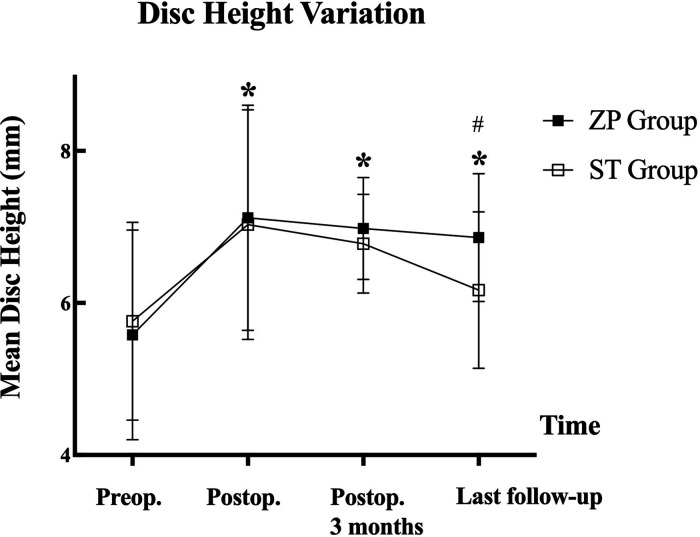
Changes of mean cervical disc height during the follow-up for ZP group and ST group. ZP, zero-P cage; ST, stand-alone cage; Preop, preoperative; Postop, postoperative. *P < 0.05, compared with preoperative value; #*P* < 0.05 significant difference between the two groups.

The descriptive statistics illustrating the DHI are listed in Table 4. After the procedure, the DHI of the treated segments recovered considerably, and there was a significant difference between the two groups at the last follow-up (Table 4). The average DHI declined steadily in both groups after surgery, but it dropped more rapidly and dramatically in the ST group, resulting in a significant difference in DHI at the last follow-up (*P* = 0.002) (Figure 4).

The fusion rates were 71/78 (91.02%) in the ZP group, and 69/76 (90.79%) in the ST group at the final follow-up (*P *> 0.05) (Table 4).

### Complications: Subsidence, dysphagia and axial cervical discomfort

The descriptive statistics illustrating other complications are listed in Table 5. Dysphagia (*n* = 1), axial cervical discomfort (*n* = 1), and subsidence (*n* = 7) occurred in the ST group, while dysphagia (*n* = 6), axial cervical discomfort (*n* = 1), and subsidence (*n* = 6) occurred in the ZP group. Although postoperative consequences were comparable between the ZP and ST groups, we detected a substantial difference in transitory postoperative dysphagia (<3 months) (*P* = 0.108) (15.4% vs. 2.6%, respectively). Just one individual in the ZP group reported symptoms of dysphagia that lasted longer than 3 months, but the symptoms were mild.

**Table 5:** Patients’ complications.

Complication	ZP group (*n* = 39)	ST group (*n* = 38)	*P* value
Dysphagia			
Postoperative	6 (15.4%)	1 (2.6%)	0.108
Final follow-up	1 (2.6%)	0	0.999
Axial neck pain	1 (2.6%)	1 (2.6%)	0.999
Subsidence	6/78 (7.7%)	7/76 (9.5%)	0.735

ST, stand-alone cages; ZP, zero-profile cages.

## Discussion

ACDF, a gold standard for patients’ refractory to non-operative treatment, has shown efficacy to treat degenerative spine conditions (16, 17). Moreover, the ZP cage and ST cage are emphatic devices, with efficacy in disc reconstruction (15). Despite being technically easier and probably evading the risk factors of ACP, the use of ST and ZP cages in two-level ACDF has been associated with multiple intraoperative and postoperative complications. For example, decreased rigidity in extension, an increased risk of vertebral subsidence, and pseudoarthrosis have been reported (1, 15, 18). We documented the clinical and radiographic findings in two-level ACDF using the ZP and ST cages. Furthermore, we compared ZP and ST cages to clarify whether the ZP cage could achieve solid fusion and keep postsurgical clinical outcomes. Our results indicated that both groups had substantial improvements in VAS, NDI, and JOA ratings in the postoperative period, suggesting that ZP and ST could contribute to equal recovery of functional status, which is in accordance with previous evidence (19).

Cervical curvature is known to contribute to excellent clinical outcomes (10). After surgery, subsidence and the size of the cage may all have an impact on cervical curvature. A larger cage increases the intervertebral pressure and provides better axial stability of the cage, thus affecting the postoperative Cobb angle (20, 21). Nakanishi et al. (16) suggested that subsidence of the spacer affected the focal angle but did not alter the C2–C7 angle or the tilt angle. In our study, the ST and ZP groups both showed a substantial increase in the degree of cervical lordosis at the final follow-up, while both groups had substantial improvements in VAS, NDI, and JOA ratings in the postoperative period. In addition, a biomechanical study showed that cages with fixation provided greater structural strength than ST spacers for two and three levels, whereas single-level fixation provided equal stiffness (19, 22, 23). This may be the reason why there were variations in radiological measurements between single-level and mixed-level with ZP and ST cages.

The better clinical recovery was substantially more obvious in solid fusion, a condition that avoids delayed kyphotic deformity. For one-segment ACDF, several studies have found similar clinical outcomes in neurological recovery and fusion regardless of whether a ZP or ST cage was used (5, 24, 25). For two-segment ACDF, nine clinical studies included in a meta-analysis revealed that the usage of a ZP cage was associated with a 90% fusion rate after 36 months of follow-up (26). According to Sun et al. (10), radiological fusion was detected in 92.59% of the cases in the ZP group after a year, and all patients of the ZP group had complete fusion after 5 years. According to multi-level research by Li et al. (5), the usage of a ST cage resulted in a fusion rate of 91.7%. In our research, the ZP group had a higher fusion rate than the ST group, but the variations between the groups were too minor to be practically meaningful.

Subsidence occurs when the measured height at any of the two-disc heights decreases by more than 2 mm. Recent statistics reveal that postsurgical subsidence occurs most frequently within the first 3 months after the treatment (27, 28). The gap under the disc surface might decline due to natural or pathological processes during the fusing process. In general, the estimated range of the percentage of patients with subsidence following ACDF is between 0% and 40% (2, 29, 30). Bartels et al. (27) reported a subsidence rate of 19.1% after the usage of ZP cage. Wu et al. (18) reported a subsidence rate of up to 29.2% after utilizing ST cages. Even when the cage subsided, Opsenak et al. (28) showed that subsidence had no serious influence on the clinical outcome. A number of additional trials have shown comparable results (2, 30, 31). In our study, the incidence of interbody fusion cage subsidence in the ZP and ST groups was 7.7% and 9.5%, respectively. A higher rate of subsidence may explain the differential disc height loss in the ST group. Although the loss of disc height significantly differed between the groups, the patients’ mid-long-term clinical outcomes were, surprisingly, still rather excellent in both groups. Additionally, the disc height in patients with subsidence was substantially higher than before. Progressive subsidence of the cage might be caused by surgery, such as cervical fusion, as well as by the movement caused by the natural procedure itself, and poor bone mineral density.

Dysphagia is a frequently reported complaint of patients after ACDF; however, it is often temporary and self-limiting (10). Persistent and chronic dysphagia poses substantial medical nutritional concerns and may increase mortality. In recent studies (32, 33), it has been shown that dysphagia was more common in the ZP group than in the ST group. The frequency of post-ACDF dysphagia varies from 0% to 76% (34, 35), likely depending on how thoroughly investigators check for dysphagia following surgery. According to a prior study, there is a notable incidence of postoperative dysphagia (40.7%) and chronic dysphagia (3.7%) after ACDF with a ZP cage (10). The occurrence of dysphagia in our study is similar to that reported in the literature (2, 36, 37). The ST group showed a lower incidence of dysphagia both in the early postoperative period (<3 months) and at the final follow-up than the ZP group. It may becasuse the removal of the anterior plating in ST group reduces the irritation to the anterior cervical tissue and esophagus. It has been proved that the thickness of the cervical plate is related to dysphagia. Therefore, the ZP cage might not be a strategy to diminish dysphagia, which agrees with the reports by Albanese and Scholz. According to Albanese et al. (32), the prevalence of dysphagia was 20.8%; likewise, according to Scholz et al. (33), among patients with a ZP cage, 15% (3/20) reported a history of mild dysphagia. When compared with the documented occurrence of chronic dysphagia following placement of the ZP cage (33, 36), our occurrence of this condition was somewhat low. A variety of variables have been implicated in the development of dysphagia following ACDF, but the exact reasons remain unknown. According to prior research, dysphagia is more common with older age (3, 38), unfavorable postoperative C2–C7 angle (39), pre-pneumonectomy (40), two-level surgery (9, 41), long operation time (36), and greater prevertebral soft tissue swelling (9, 42). Further research on dysphagia mechanisms and techniques for reducing the occurrence of dysphagia are necessary.

The current study is limited by its retrospective nature and small sample size. Besides, the follow-up time was not long enough to demonstrate the mid-long-term impact of the ZP and ST cages for cervical degenerative disc disease. However, given there are few papers describing outcomes after ZP and ST cages for two-level ACDF, we believe this study provides helpful information about surgical intervention for cervical radiculopathy and myelopathy. To overcome the limitations and identify whether the ZP cage is better than the ST cage for ACDF, larger and prospective, randomized studies with long-term follow-up periods are needed.

## Conclusion

ACDF with ZP cages is an effective and safe procedure for two-level cervical spondylosis. Compared with ST cages, ZP cages generate less disc height loss and fewer negative issues associated with cervical curvature.

## Data Availability

The original contributions presented in the study are included in the article/Supplementary Material, further inquiries can be directed to the corresponding author/s.

## References

[1] Li Z Zhao Y Tang J Ren D Guo J Wang H A comparison of a new zero-profile, stand-alone fidji cervical cage and anterior cervical plate for single and multilevel ACDF: a minimum 2-year follow-up study. Eur Spine J. (2017) 26(4):1129–39. 10.1007/s00586-016-4739-2 27554353

[2] Lu Y Bao W Wang Z Zhou Z Zou F Jiang J Yang W Comparison of the clinical effects of zero-profile anchored spacer (ROI-C) and conventional cage-plate construct for the treatment of noncontiguous bilevel of cervical degenerative disc disease (CDDD): a minimum 2-year follow-up. Medicine. (2018) 97(5):e9808. 10.1097/md.0000000000009808 29384883 PMC5805455

[3] Smith-Hammond CA New KC Pietrobon R Curtis DJ Scharver CH Turner DA. Prospective analysis of incidence and risk factors of dysphagia in spine surgery patients: comparison of anterior cervical, posterior cervical, and lumbar procedures. Spine. (2004) 29(13):1441–6. 10.1097/01.brs.0000129100.59913.ea 15223936

[4] Vaishnav AS Saville P McAnany S Patel D Haws B Khechen B Predictive factors of postoperative dysphagia in single-level anterior cervical discectomy and fusion. Spine. (2019) 44(7):E400–E07. 10.1097/brs.0000000000002865 30889144 PMC11296389

[5] Li Z Wang H Li L Tang J Ren D Hou S. A new zero-profile, stand-alone fidji cervical cage for the treatment of the single and multilevel cervical degenerative disc disease. J Clin Neurosci. (2017) 41:115–22. 10.1016/j.jocn.2017.02.043 28262396

[6] Fayed I Conte AG Keating G Cobourn KD Altshuler M Makariou E Comparison of clinical and radiographic outcomes after standalone versus cage and plate constructs for anterior cervical discectomy and fusion. Int J Spine Surg. (2021) 15(3):403–12. 10.14444/8060 33963034 PMC8176849

[7] Fujibayashi S Neo M Nakamura T. Stand-alone interbody cage versus anterior cervical plate for treatment of cervical disc herniation: sequential changes in cage subsidence. J Clin Neurosci. (2008) 15(9):1017–22. 10.1016/j.jocn.2007.05.011 18653347

[8] Li N Wang R Teng W Yu J. Zero-profile versus cage-plate interbody fusion system in anterior cervical discectomy and fusion for the treatment of multilevel cervical spondylosis: a protocol of systematic review and meta-analysis. Medicine. (2020) 99(35):e22026. 10.1097/md.0000000000022026 32871958 PMC7458262

[9] Huang C Abudouaini H Wang B Ding C Meng Y Yang Y Comparison of patient-reported postoperative dysphagia in patients undergoing one-level versus two-level anterior cervical discectomy and fusion with the zero-P implant system. Dysphagia. (2021) 36(4):743–53. 10.1007/s00455-020-10197-w 33387002

[10] Sun B Shi C Wu H Xu Z Lin W Shen X Application of zero-profile spacer in the treatment of three-level cervical spondylotic myelopathy: 5-year follow-up results. Spine. (2020) 45(8):504–11. 10.1097/brs.0000000000003312 32224806

[11] Virkar N Bhilare P Hadgaonkar S Kothari A Sancheti P Aiyer S. Standalone cage versus anchored cage for anterior cervical discectomy and fusion: a comparative analysis of clinical and radiological outcomes. Int Orthop. (2022) 46(10):2339–45. 10.1007/s00264-022-05493-z 35790547

[12] Hoppenbrouwers M Eckhardt MM Verkerk K Verhagen A. Reproducibility of the measurement of active and passive cervical range of motion. J Manipulative Physiol Ther. (2006) 29(5):363–7. 10.1016/j.jmpt.2006.04.007 16762663

[13] Sakai K Yoshii T Arai Y Hirai T Torigoe I Inose H A prospective cohort study of dysphagia after subaxial cervical spine surgery. Spine. (2021) 46(8):492–98. 10.1097/brs.0000000000003842 33306616

[14] Li Z Yu S Zhao Y Hou S Fu Q Li F Clinical and radiologic comparison of dynamic cervical implant arthroplasty versus anterior cervical discectomy and fusion for the treatment of cervical degenerative disc disease. J Clin Neurosci. (2014) 21(6):942–8. 10.1016/j.jocn.2013.09.007 24411326

[15] Gerszten PC Paschel E Mashaly H Sabry H Jalalod’din H Saoud K. Outcomes evaluation of zero-profile devices compared to stand-alone PEEK cages for the treatment of three- and four-level cervical disc disease. Cureus. (2016) 8(9):e775. 10.7759/cureus.775 27738574 PMC5059158

[16] Nakanishi Y Naito K Yamagata T Masaki Yoshimura Shimokawa N Nishikawa M Safety of anterior cervical discectomy and fusion using titanium-coated polyetheretherketone stand-alone cages: multicenter prospective study of incidence of cage subsidence. J Clin Neurosci. (2020) 74:47–54. 10.1016/j.jocn.2020.01.056 31983642

[17] Pereira EA Chari A Hempenstall J Leach JC Chandran H Cadoux-Hudson TA. Anterior cervical discectomy plus intervertebral polyetheretherketone cage fusion over three and four levels without plating is safe and effective long-term. J Clin Neurosci. (2013) 20(9):1250–5. 10.1016/j.jocn.2012.10.028 23890411

[18] Wu WJ Jiang LS Liang Y Cage subsidence does not, but cervical lordosis improvement does affect the long-term results of anterior cervical fusion with stand-alone cage for degenerative cervical disc disease: a retrospective study. Eur Spine J. (2012) 21(7):1374–82. 10.1007/s00586-011-2131-9 22205113 PMC3389116

[19] Scholz M Schleicher P Pabst S Dai LY. A zero-profile anchored spacer in multilevel cervical anterior interbody fusion: biomechanical comparison to established fixation techniques. Spine. (2015) 40(7):E375–80. 10.1097/brs.0000000000000768 25584947

[20] Igarashi H Hoshino M Omori K Matsuzaki H Nemoto Y Tsuruta T Factors influencing interbody cage subsidence following anterior cervical discectomy and fusion. Clin Spine Surg. (2019) 32(7):297–302. 10.1097/bsd.0000000000000843 31169615

[21] Shi S Liu ZD Li XF Qian L Zhong GB Chen FJ Comparison of plate-cage construct and stand-alone anchored spacer in the surgical treatment of three-level cervical spondylotic myelopathy: a preliminary clinical study. Spine J. (2015) 15(9):1973–80. 10.1016/j.spinee.2015.04.024 25912505

[22] Kinon MD Greeley SL Harris JA Gelfand Y Yassari R Nakhla J Biomechanical evaluation comparing zero-profile devices versus fixed profile systems in a cervical hybrid decompression model: a biomechanical in vitro study. Spine J. (2020) 20(4):657–64. 10.1016/j.spinee.2019.10.004 31634616

[23] Paik H Kang DG Lehman RA Cardoso MJ Gaume RE Ambati DV Do stand-alone interbody spacers with integrated screws provide adequate segmental stability for multilevel cervical arthrodesis? Spine J 2014;14(8):1740–7. 10.1016/j.spinee.2014.01.034 24462812

[24] Li T Yang JS Wang XF Meng CY Wei JM Wang YX Can zero-profile cage maintain the cervical curvature similar to plate-cage construct for single-level anterior cervical diskectomy and fusion? World Neurosurg. (2020) 135:e300–e06. 10.1016/j.wneu.2019.11.153 31805404

[25] Noh SH Zhang HY. Comparison among Perfect-C®, Zero-P®, and plates with a cage in single-level cervical degenerative disc disease. BMC Musculoskelet Disord. (2018) 19(1):33. 10.1186/s12891-018-1950-9 29368613 PMC5784656

[26] Lu VM Mobbs RJ Fang B Phan K. Clinical outcomes of locking stand-alone cage versus anterior plate construct in two-level anterior cervical discectomy and fusion: a systematic review and meta-analysis. Eur Spine J. (2019) 28(1):199–208. 10.1007/s00586-018-5811-x 30390163

[27] Bartels RH Donk RD Feuth T. Subsidence of stand-alone cervical carbon fiber cages. Neurosurgery. (2006) 58(3):502–8; discussion 2–8. 10.1227/01.Neu.0000197258.30821.50 16528190

[28] Opsenak R Hanko M Snopko P Varga K Kolarovszki B. Subsidence of anchored cage after anterior cervical discectomy. Bratisl Lek Listy. (2019) 120(5):356–61. 10.4149/bll_2019_058 31113198

[29] Lonjon N Favreul E Huppert J Lioret E Delhaye M Mraidi R Clinical and radiological outcomes of a cervical cage with integrated fixation. Medicine. (2019) 98(3):e14097. 10.1097/md.0000000000014097 30653129 PMC6370175

[30] Ng EP Yip AS Wan KH Tse MS Wong KK Kwok TK Stand-alone cervical cages in 2-level anterior interbody fusion in cervical spondylotic myelopathy: results from a minimum 2-year follow-up. Asian Spine J. (2019) 13(2):225–32. 10.31616/asj.2018.0193 30472820 PMC6454285

[31] Shiban E Nies M Kogler J Kogler L da Cunha PR Meyer B No correlation between radiological and clinical outcome 1 year following cervical arthrodesis. Acta Neurochir. (2018) 160(4):845–53. 10.1007/s00701-018-3495-y 29479658

[32] Albanese V Certo F Visocchi M Barbagallo G. Multilevel anterior cervical diskectomy and fusion with zero-profile devices: analysis of safety and feasibility, with focus on sagittal alignment and impact on clinical outcome: single-institution experience and review of literature. World Neurosurg. (2017) 106:724–35. 10.1016/j.wneu.2017.06.051 28625909

[33] Scholz M Onal B Schleicher P Pingel A Hoffmann C Kandziora F Two-level ACDF with a zero-profile stand-alone spacer compared to conventional plating: a prospective randomized single-center study. Eur Spine J. (2020) 29(11):2814–22. 10.1007/s00586-020-06454-z 32430769

[34] Hofstetter CP Kesavabhotla K Boockvar JA. Zero-profile anchored spacer reduces rate of dysphagia compared with ACDF with anterior plating. J Spinal Disord Tech. (2015) 28(5):E284–90. 10.1097/BSD.0b013e31828873ed 23429316

[35] Yang Y Ma L Liu H Xu M. A meta-analysis of the incidence of patient-reported dysphagia after anterior cervical decompression and fusion with the zero-profile implant system. Dysphagia. (2016) 31(2):134–45. 10.1007/s00455-015-9681-7 26753930

[36] Lu Y Fang Y Shen X Lu D Zhou L Gan M Zhu X. Does zero-profile anchored cage accompanied by a higher postoperative subsidence compared with cage-plate construct? A meta-analysis. J Orthop Surg Res. (2020) 15(1):189. 10.1186/s13018-020-01711-9 32448320 PMC7247200

[37] Zhu D Zhang D Liu B Li C Zhu J. Can self-locking cages offer the same clinical outcomes as anterior cage-with-plate fixation for 3-level anterior cervical discectomy and fusion (ACDF) in mid-term follow-up? Med Sci Monit. (2019) 25:547–57. 10.12659/msm.911234 30659165 PMC6347916

[38] Yang Y Ma L Liu H Liu Y Hong Y Wang B Comparison of the incidence of patient-reported post-operative dysphagia between ACDF with a traditional anterior plate and artificial cervical disc replacement. Clin Neurol Neurosurg. (2016) 148:72–8. 10.1016/j.clineuro.2016.07.020 27428486

[39] Huang CY Meng Y Wang BY Yu J Ding C Yang Y The effect of the difference in C(2-7) angle on the occurrence of dysphagia after anterior cervical discectomy and fusion with the zero-P implant system. BMC Musculoskelet Disord. (2020) 21(1):649. 10.1186/s12891-020-03691-7 33023551 PMC7539444

[40] Fisahn C Schmidt C Rustagi T Moisi M Iwanaga J Norvell DC Comparison of chronic dysphagia in standalone versus conventional plate and cage fusion. World Neurosurg. (2018) 109:e382–e88. 10.1016/j.wneu.2017.09.188 28987856

[41] Fengbin Y Xinwei W Haisong Y Dysphagia after anterior cervical discectomy and fusion: a prospective study comparing two anterior surgical approaches. Eur Spine J. (2013) 22(5):1147–51. 10.1007/s00586-012-2620-5 23277296 PMC3657041

[42] Min Y Kim WS Kang SS Yu C Xiaowei L Deyu C. Incidence of dysphagia and serial videofluoroscopic swallow study findings after anterior cervical discectomy and fusion: a prospective study. Clin Spine Surg. (2016) 29(4):E177–81. 10.1097/bsd.0000000000000060 24326242

